# The Aha! experience is associated with a drop in the perceived difficulty of the problem

**DOI:** 10.3389/fpsyg.2024.1314531

**Published:** 2024-01-23

**Authors:** Nadezhda V. Moroshkina, Elena I. Pavliuchik, Artur V. Ammalainen, Valeria A. Gershkovich, Olga V. Lvova

**Affiliations:** ^1^Institute for Cognitive Studies, St. Petersburg University, St. Petersburg, Russia; ^2^Institute of Psychology, University of Greifswald, Greifswald, Germany

**Keywords:** insight, Aha! experience, subjective difficulty, processing fluency, metacognitive prediction error, rebus puzzles

## Abstract

The study investigated the correlation between the intensity of the Aha! experience and participants’ subjective difficulty ratings of problems before and after finding their solutions. We assumed that the Aha! experience arises from a shift in processing fluency triggered by changing from an initially incoherent problem representation to a coherent one, which ultimately leads to the retrieval of a solution with unexpected ease and speed. First, we hypothesized that higher Aha! experience ratings would indicate more sudden solutions, manifesting in a reduced correlation between the initial difficulty ratings and solution times. Second, we hypothesized that higher Aha! experience ratings would correspond to a greater shift in the subjective difficulty ratings between the initial and retrospective assessments. To test our hypotheses, we developed a novel set of rebus puzzles. A total of 160 participants solved rebuses and provided initial (within 5 s of problem presentation) and retrospective difficulty ratings (following the generation or presentation of a correct solution). They also rated their Aha! experience (after solution generation or presentation), confidence in solutions, and the likability of each rebus. Our findings revealed that the initial ratings of the problem’s subjective difficulty were positively correlated with the solution time and that this correlation decreased in the case of a stronger Aha! experience. Aha! experience ratings were positively correlated with the differences between initial and retrospective difficulty ratings, confidence, solution accuracy, and rebus likability. We interpreted our results to be in line with the processing fluency and metacognitive prediction error accounts.

## 1 Introduction

Problem-solving and creative thinking are important aspects of everyday life and many professional activities. It is commonly accepted that problem-solving occurs via analytical processing (step-by-step mode) or by sudden restructuring of mental representations (insight mode; Sternberg and Davidson, 1995). Insight solutions are accompanied by an Aha! experience, which is considered an affective component of insight (Duncker, 1945; Gick and Lockhart, 1995; Danek et al., 2014). When solvers employ an analytical approach to problem-solving, they can usually report the intermediate steps that led them to the solution. In contrast, with insight solutions, people report suddenly finding the solution. Metcalfe and Wiebe (1987) first proposed the use of warmth ratings as a metric of subjective progress during problem-solving, showing that while the warmth ratings in algebraic problems gradually increased toward the solution, in classical insight problems, the ratings remained low and increased immediately before the solution. Subsequent studies on the subjective suddenness of insight revealed similar results using various problems (Kizilirmak et al., 2018; Laukkonen et al., 2021). For example, Kizilirmak et al. (2018) provided participants with compound remote associate test (CRAT) problems, which consist of three seemingly unrelated clue words. The solvers have to find a target word that forms a compound word with each of the clue words. In the study of Kizilirmak et al. (2018), participants indicated how close they were to the solution using warmth rating during the solving process and reported whether they had an Aha experience after finding the solution. The results revealed a correlation between two measures: solutions with Aha corresponded to a discontinuous pattern of warmth ratings while solutions without Aha were connected to a more continuous one (Kizilirmak et al., 2018). However, some research showed mixed results (for more detail, see Hedne et al., 2016; Laukkonen and Tangen, 2018).

Scholars have proposed various approaches to explain cognitive mechanisms leading to a subjectively sudden solution. Most researchers agree that the representational change is the key event of an insightful solution (Knoblich et al., 1999; Kounios and Beeman, 2014; Weisberg, 2015) but propose different approaches to explain it. According to the first approach, the process of problem-solving occurs at an unconscious level for some time, and then a solution comes to mind all at once, without any intermediate stages (Bowden et al., 2005; Ohlsson, 2011; Kounios and Beeman, 2014; Olteţeanu and Falomir, 2015; Valueva and Ushakov, 2017). In other words, the lack of conscious access to the unfolding cognitive processes is the reason for subjectively sudden solutions. For example, several demonstrated that unconscious cues can trigger insightful solutions (Bowden, 1997; Ammalainen and Moroshkina, 2022).

In the alternative approach, the solution process is fully conscious, and the Aha! experience occurs because solvers fail to predict the outcome of a representational change (Kaplan and Simon, 1990; Simon, 1995; Weisberg, 2015). It is assumed that, while solving a problem in the first stage, the solver forms an erroneous or an incomplete representation of the problem space. Within that space, the search for a solution is fruitless. However, as the number of failed attempts accumulates, the solver gains additional information about the problem that provokes them to switch to searching for another representative space (Kaplan and Simon, 1990). If the solution is reached in one or two steps in this new problem space (in other words, it falls within the “horizon of mental lookahead”; Ohlsson, 1984), it will be subjectively sudden and experienced as an insight. As Gick and Lockhart (1995) emphasize in their work, the very process of finding a new representation can be long and painful. However, when an appropriate representation is found, the answer is retrieved from memory automatically, quickly, and effortlessly, which causes the experience of suddenness.

Subjective reports on solution suddenness are often used to prove the special status of cognitive processes leading to insights (Metcalfe and Wiebe, 1987; Stuyck et al., 2023), and an Aha! experience is considered a marker of these processes. In the last two decades the phenomenology of Aha! experience itself became a subject of interest for researchers (see, for example, Topolinski and Reber, 2010; Danek et al., 2014; Skaar and Reber, 2020; Danek, 2023). However, the mechanisms underlying the occurrence of Aha! experience remain unclear. Why are some solutions to problems accompanied by an Aha! experience while others are not? What determines the intensity of the Aha! experience?

Research on the phenomenology of insight has revealed that the Aha! experience is a multi-dimensional phenomenon: in addition to experiencing the suddenness of the solution, it includes confidence in the correctness of the solution and positive emotions (Danek and Wiley, 2017). Various characteristics (e.g., surprise, certainty, happiness, drive, and relief) are described as Aha! experience components (Danek et al., 2014; Shen et al., 2016; Danek and Wiley, 2017; Webb et al., 2019; Stuyck et al., 2021). Moreover, several studies have shown that solutions accompanied by Aha! experiences are more likely to be correct, the so-called “Aha!-accuracy effect” (Salvi et al., 2016a; Danek and Salvi, 2020). However, some authors claim that the Aha!-accuracy effect reflects the specificity of the problems used to study insights and is not necessarily observed outside the laboratory (Webb et al., 2019; Strickland et al., 2022).

Topolinski and Reber (2010) suggested that the Aha! experience is triggered by an abrupt increase in the processing fluency of a problem at the moment of solution discovery (i.e., the processing fluency approach). Processing fluency is usually understood as the ease and/or speed with which information is processed in a cognitive system, as well as the degree of the representation’ coherence (Winkielman et al., 2012). Experiencing the processing fluency as a byproduct of a wide array of cognitive processes, people interpret it as a cue for various metacognitive judgments (e.g., a judgment of the familiarity of the stimulus, confidence in the solution; Alter and Oppenheimer, 2009, or of the solvability of the problem; Topolinski and Strack, 2008). Reber and Schwarz (1999) showed that an increase in processing fluency can provoke a truth effect (i.e., confidence in the reliability of the presented information). Other studies have also demonstrated that increased processing fluency itself is experienced as a positive affect (Winkielman et al., 2003; Reber et al., 2004). Thus, the concept of processing fluency provides a common explanation for such characteristics of the Aha! experience as confidence in the solution’s correctness, positive emotions, and the feeling that a solution came effortlessly.

Dubey et al. (2021) proposed that the Aha! experience is associated with a metacognitive prediction error and occurs when the time taken to solve a problem turns out to be less than was predicted in the early stages of problem-solving. They consider the Aha! experience to be an internal reward (a positive reward prediction error), thus explaining why positive emotions are an essential component of the Aha! experience.

From our perspective, the approaches mentioned above are similar since they link the Aha! experience with the functioning of the metacognitive system. People utilize metacognitive monitoring to assess their chances of success before, during, and after performing a cognitive task, and they use these judgments to allocate their mental efforts, obtain assistance, etc. (Ackerman and Thompson, 2017; Ackerman, 2019). The main approach in metacognition research—the cue utilization approach—assumes that people do not reliably know their knowledge level but infer it based on heuristics (Koriat, 1997). Processing fluency is one of the main cues. It has been shown that when facing a problem, a solver forms an initial judgment of its solvability based on the processing fluency heuristic (Topolinski and Strack, 2008), and this judgment, in some cases, predicts the probability and time needed to solve the problem (Lauterman and Ackerman, 2019; Burton et al., 2023). However, the relationship between the initial judgment of solvability/difficulty ratings of a problem with the probability of gaining the insightful solution, time taken to solve it and the intensity of the corresponding Aha! experience remains unclear.

In our previous study (Moroshkina et al., 2022), we developed approach of Topolinski and Reber (2010). Specifically, we suggested that when introduced to a problem, a solver intuitively assesses its difficulty based on the processing fluency of the primary representation and forms the corresponding expectations about the time and cognitive resources that must be allocated to solve it. As a result of restructuring, the primary representation is replaced by a new one. If the new representation is more coherent, this leads to a more fluent (faster) solution retrieval than expected. This, in turn, triggers the Aha! experience, and the assessment of the problem’s difficulty is corrected downward. This assumption is close to the idea expressed by Dubey et al. (2021). However, our idea explains not only the process of solution generation (endogenous insights) but also cases of sudden understanding of the presented solution (induced insights), when participants report an Aha! experience after being introduced to the correct answer (see, e.g., Kizilirmak et al., 2016, 2021; Moroshkina et al., 2022).

Thus, the described approaches suggest that the probability and intensity of the Aha! experience is closely related to the metacognitive assessments of the problem itself. As we described above, if an abrupt increase in processing fluency underlies the Aha! experience, it should manifest in a decrease in the perceived difficulty of the problem, be it after finding the solution or after becoming familiar with it. In a previous study, we examined this hypothesis and found the confirmation only for the latter case that is for the solution presentation’s condition (Moroshkina et al., 2022). Participants completed remote association tasks and upon completion reported whether they had had an Aha! experience and whether the problem had seemed difficult. The Aha! experience was rated in two different situations: (1) after successful solution generation (post-solution-generation Aha! experiences) and (2) in the case of an unsuccessful generation, after the presentation of the answer (post-solution-presentation Aha! experiences). The results showed that post-solution-presentation Aha! experience was associated with a decreased likelihood of judging problems as difficult. We called this effect “difficulty estimation bias.” This result was obtained with the control for the problem’s objective difficulty calculated as the proportion of correct solutions to each problem, aggregated for the entire sample. Based on this result, we supposed that the post-solution-presentation Aha! experience resembles the feeling that “I knew it all along!” (Fischhoff, 1977; Hasher et al., 1981). Participants felt that the problem was not difficult, and they could solve it. Such cases were previously described in the work of Gick and Lockhart (1995, p. 199): some of their participants even exclaimed: “I’ve been duped!” or “Why did not I think of that before?”

In an independent study, Stuyck et al. (2021) also investigated the association of the Aha! experience with the problem’s subjective difficulty using CRAT material. In their work, participants assessed the difficulty of the problem twice: (1) within 2 s after introducing the problem and (2) after discovering the solution. Both ratings were made using a visual analog scale that ranged from red (difficult = 0) to green (easy = 100). The authors found solutions with the Aha! experience were more common for problems that were initially rated as more difficult. Based on the assumption that the Aha! experience reflects metacognitive prediction error, we might expect that in the study of Stuyck et al. (2021), the subjective difficulty of a problem solved with a stronger Aha! experience should have been significantly reduced when the problem difficulty was reassessed after the solution. Thus, we conducted an additional analysis of data of Stuyck et al. (2021)^1^ and confirmed the aforementioned assumption: the decline in difficulty ratings was more significant for problems solved with the Aha! experience than for those solved without the Aha! experience (for more details, see Moroshkina et al., 2022, p.10).

### 1.1 The current study

This study aimed to investigate the connection between the Aha! experience and change in the subjective difficulty assessments of the problem before and after finding the solution or solution presentation. Based on the approaches of Topolinski and Reber (2010) and Dubey et al. (2021), as well as previous studies (Stuyck et al., 2021; Moroshkina et al., 2022), we hypothesized that the intensity of the Aha! experience would reflect the magnitude of the unexpected processing fluency gain achieved as a result of restructuring and solution retrieval. If such, the assessment of the Aha! experience should correlate with the magnitude of the difference in the subjective assessments of the problem’s difficulty before and after the solution. In other words, in the early stages of problem-solving, the participant will assess the problem as difficult, predicting a longer time and greater allocation of mental efforts to solve it. However, as a result of the restructuring, the solution suddenly enters the participant’s consciousness with greater ease and speed than expected, thus inducing the Aha! experience. Therefore, we also assumed that the problem’s subjective difficulty ratings given after introducing the problem would be more strongly correlated with the search time for non-insightful solutions than for insightful ones (with the Aha! experience). This effect will reflect the connection of the Aha! experience to the metacognitive prediction error.

Previous studies have investigated the connection between the Aha! experience and ratings of subjective problem difficulty on fairly simple verbal tasks [anagrams (Dubey et al., 2021) and CRAT/RAT (Stuyck et al., 2021; Moroshkina et al., 2022)]. Our study expands our understanding of the connection between the Aha! experience and metacognitive assessments as we use rebus puzzles and analyze Aha! ratings after both solution generation and solution presentation. Previous studies have shown that rebus puzzles are suitable materials for insight research. However, rebus databases have only been developed in the English (MacGregor and Cunningham, 2008; Threadgold et al., 2018) and Italian languages (Salvi et al., 2016b). For this study, we developed a set of rebus puzzles in Russian that is similar to previously published databases (for more details, see the section “Materials and methods”).

We used Likert scales to measure the intensity of the Aha! experience and the subjective difficulty of the problem. The Aha! experience was assessed in two different situations: (1) after a successful solution generation (post-solution-generation Aha! experiences) and (2) in the case of an unsuccessful generation, after the presentation of the answer (post-solution-presentation Aha! experiences). The subjective difficulty of the problem was assessed three times: (1) immediately after the problem was introduced [the initial difficulty rating (dif1)], (2) after the generation of the solution [the second retrospective difficulty rating (dif2)], and (3) if the solution was incorrect or it was not found within the allotted time, after the presentation of the correct answer [third retrospective difficulty rating (dif3)].

The main hypotheses are as follows:

1. Initial ratings of the problem’s subjective difficulty positively correlate with the solution time of the problem, but the strength of this correlation decreases with an increase in Aha! experience ratings.
2. The Aha! experience ratings are positively correlated with the difference between subjective difficulty ratings before and after understanding the solution (when controlling the solution time).
  - Post-solution-generation Aha! experience ratings are positively correlated with the difference between initial difficulty ratings and the second retrospective difficulty ratings.
  - Post-solution-presentation Aha! experience ratings are positively correlated with the difference between initial difficulty ratings and the third retrospective difficulty ratings.

Since we developed a new set of problems—polycode rebus puzzles in Russian—we also examined the extent to which the phenomenological characteristics of the Aha! experience that accompany the solution to this type of problems corresponded to the results of previous studies, especially those that used similar materials (Salvi et al., 2016b; Threadgold et al., 2018). Numerous studies have shown that the Aha! experience correlates with high confidence in the correctness of the solution (Danek and Wiley, 2017), solution accuracy (Salvi et al., 2016a,b; Threadgold et al., 2018), positive emotions (Danek et al., 2014; Shen et al., 2016; Danek and Wiley, 2017; Savinova and Korovkin, 2022), and a positive evaluation of the problem; that is, likability (Moroshkina et al., 2022), which follows the processing fluency account of Aha! experience.

Thus, we put forward the auxiliary hypotheses:

1. Post-solution-generation Aha! experience ratings are correlated with confidence in the answer (the Aha!-confidence effect hypothesis).
2. Post-solution-generation Aha! experience ratings are higher for correct solutions than for incorrect ones (the Aha!-accuracy effect hypothesis).
3. The Aha! experience ratings, both in the case of solution generation and presentation, are correlated with the assessment of rebus likability (the Aha!-likability effect hypothesis).

## 2 Materials and methods

### 2.1 Participants

The study involved 164 volunteers. We drew on two sources when planning the sample size for this study. On the one hand, we used an effect size derived from Stuyck et al. (2021) to perform an *a priori* power analysis for the relationship between the Aha! experience and the magnitude of the difference in the problem’s subjective difficulty ratings before and after obtaining its solution. The analysis was conducted using samplesize_mixed function from the sjstats R package, version 0.182 (Lüdecke, 2022) and revealed that 68 participants would be sufficient to achieve a statistical power of 0.9. However, as we tested new stimuli, which usually requires more participants. Thus, we relied on English rebus puzzle normative study of Threadgold et al. (2018) with two sets of 42 items and 170 participants.

Data from four participants were excluded from the analysis for technical reasons or instruction violations. Data from 160 participants aged 18–36 years [mean = 23.36; standard deviation (SD) = 4.6] were used for the analysis (114 females, 45 males, one identified as non-binary). All participants were native Russian speakers. Data were collected offline (94 participants) and online under the supervision of the experimenter through video communication (66 participants).

Analysis of the average accuracy performance, aggregated per participant, including gender, age, and experimental format (online or offline) factors, did not reveal any significance (ANOVA, *p* > 0.05). Therefore, all data were analyzed together.

### 2.2 Materials

Based on previous studies (MacGregor and Cunningham, 2008; Salvi et al., 2016b; Threadgold et al., 2018), we developed 114 polycode rebuses. The answers to the rebuses were common Russian expressions. Font characteristics (style, color, size, etc.), spatial arrangement, number of words, and math signs were used as various encryption principles. To solve these rebuses, it is necessary to restructure the initial mental representation: polycode rebuses require people to reinterpret the meanings of words while taking into account their visual clues. These rebuses trigger restructuring because a solver needs to refuse initial and more common interpretations and relax certain constraints (MacGregor and Cunningham, 2008; Salvi et al., 2016b).

For, example, in “ЗНА НИЯ” (“KNOW LEDGE”) the cue is the visual gap in the word that should be interpreted meaningfully (solution in Russian: “пробел в знаниях,” literally in English: “*gap in* the knowledge,” that means “knowledge gap”). The rebus “ГЛАЗА^2^” (“EYES^2^”) is solved by decoding the math sign “^2^” as a word (solution in Russian: “квадратные глаза,” literally in English: “squared eyes,” that means expression of extreme surprise or confusion). Finally, the rebus “ДНОДНО” (“BOTTOMBOTTOM”) is solved by interpreting the repetition of writing the same word (“ДНО”) twice as adjective double (two times; solution in Russian: “двойное дно,” literally in English: “double bottom,” that means a metaphor indicating the presence of a hidden meaning, a trick, or a deceptive appearance).

We used approximately 20 encryption principles to create the rebus puzzle pool. Individual rebuses included a minimum of 1 to a maximum of three principles. The 114 rebuses were divided into two main sets of 55 problems (Sets 1 and 2), with similar encoding principles balanced between sets, and one training set (three problems) and one example for the instruction. The file with all sets of rebuses can be found on the OSF.^2^ A professional graphic designer, Faina Khamidullina, illustrated the rebuses.

### 2.3 Equipment

The offline part of the experiment was carried out using PsychoPy software, version 2021.1 (Peirce et al., 2019), and the online part was conducted using PsychoPy software, version 2021.1 and Pavlovia.^3^

### 2.4 Procedure

The first part of the data was acquired offline. Participants solved problems independently on a computer. First, in line with previous research (Jung-Beeman et al., 2004; Kizilirmak et al., 2016; Moroshkina et al., 2022), we presented participants with a detailed description of the Aha! experience. The description of the Aha! experience was as follows (translation from Russian):

The Aha! experience is a feeling that you might have when the answer suddenly comes to your mind, as if out of nowhere, and it seems obvious. The most striking example of the Aha! experience, as described in the literature, is the case of Archimedes, who suddenly understood how to solve a problem and jumped out of the bath shouting “Eureka!” We do not expect that in this study you will experience the same strong feelings. However, if while solving some of the problems you experience something similar to a sudden insight, mark that you had an Aha! experience. An Aha! experience can also occur when you are presented with the correct answer without solving the problem yourself (the feeling of “Oh, exactly!”). We will ask you to assess this feeling as well. You will also rate your level of confidence in the answer, the difficulty of the rebus, and how much you liked the rebus’ idea.

**Figure 1:**
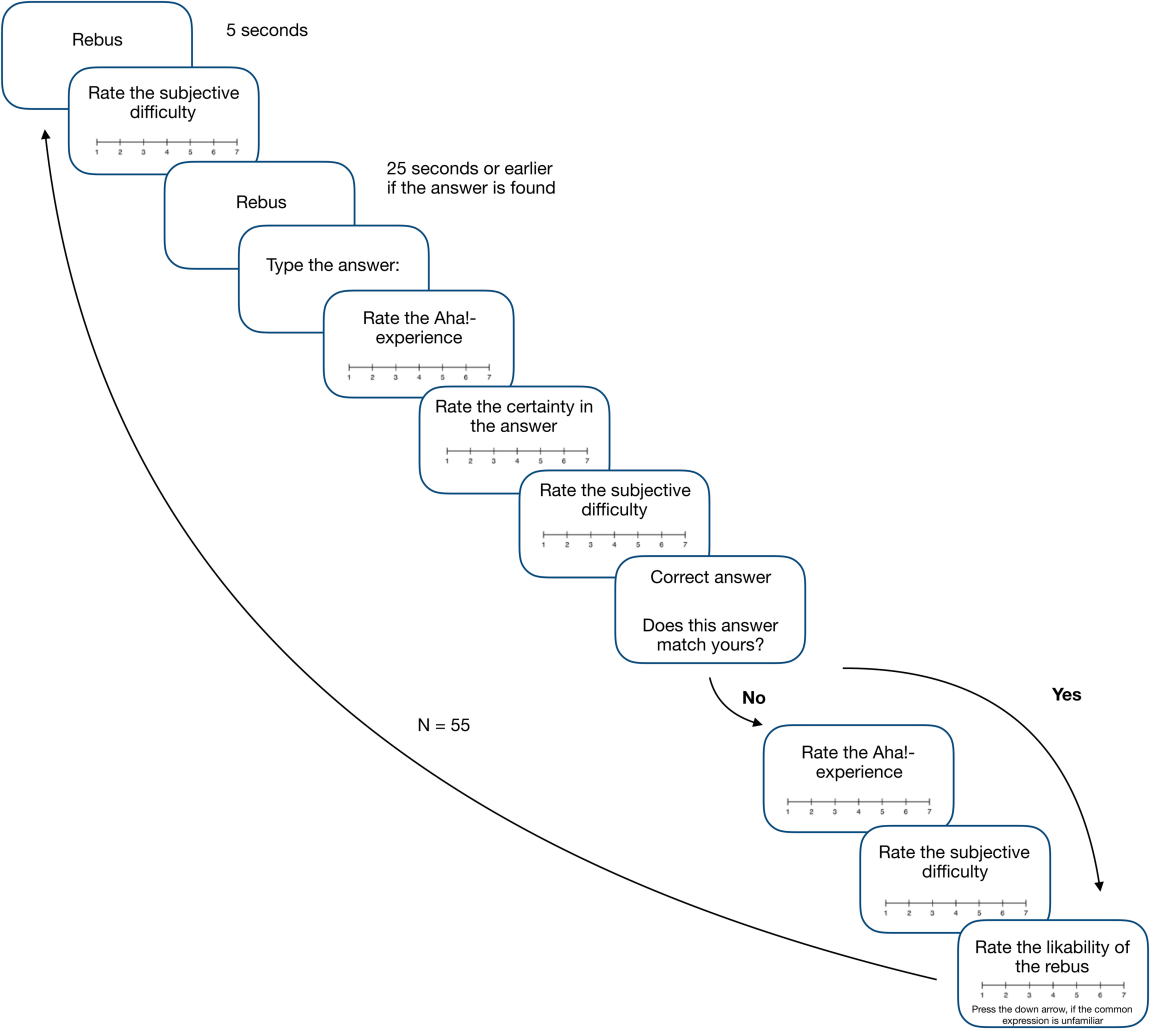
The example of a trial.

After reading the description of the Aha! experience, participants were presented with instructions on the problems to be solved during the experiment (see Supplementary Material 1). This was followed by a training phase consisting of three rebus puzzles. Then participants proceeded to the main phase, where they solved 55 rebuses (randomly assigned to Set 1 or 2). Figure 1 shows the timeline of one trial. Each rebus appeared in the center of the screen on a white background. The solution time was limited to 25 s. If participants solved the rebus before the allotted time, they pressed the SPACE BAR, and a field for entering a solution appeared.

Five seconds after the rebus presentation, the initial (first) problem’s subjective difficulty rating appeared. Participants rated the problem’s subjective difficulty on a seven-point scale (1 = It is not difficult at all, 7 = It is very difficult). After completing the assessment, the rebus reappeared again. After entering their solution using the keyboard, participants were asked to rate their Aha! experience on a seven-point scale (1 = I did not experience it at all, 7 = I had a very strong Aha! experience) by clicking on the scale. After assessing the Aha! experience, the confidence rating appeared. Participants reported their level of confidence from 1 to 7 (1 = I am not certain at all, 7 = I am very certain). Then the second assessment of rebus’ subjective difficulty appeared. Participants rated it from 1 to 7 (1 = It is not difficult at all, 7 = It is very difficult) again. If participants could not solve a rebus and did not enter any solution, these three assessments (Aha! experience, confidence, and subjective difficulty) were omitted.

Participants were then presented with the correct solution and asked to check whether their solution matched the correct solution. If it matched, the likability rating of the rebus idea appeared, and participants rated it from 1 to 7 (1 = I do not like it at all, 7 = I like it a lot). If participants’ solutions did not match the correct solution or were omitted, they were asked to rate whether they had an Aha! experience when presented with the correct solution on a seven-point scale (1 = I did not experience it at all, 7 = I had a very strong Aha! experience.). Next, they rated the subjective difficulty of the rebus after the correct solution presentation (1 = It is not difficult at all, 7 = It is very difficult). Finally, they assessed the likability of the unsolved rebus idea from 1 to 7 (1 = I do not like it at all, 7 = I like it a lot). If they did not know the common expression that was encrypted, they indicated it as unfamiliar by pressing the DOWN arrow on the keyboard. After solving all 55 rebuses, participants took part in the post-experimental interview conducted by the researcher.

The experimental program was the same in the online and offline formats. The only difference was that in the online format the experimenter observed each participant via video communication. The experimenter communicated with participants via Skype, Zoom, or Google Meet software and asked them to share their screens. Thus, the experimenter could observe all the participant’s actions. Finally, after solving all rebuses, participants were able to express general thoughts and feelings about the experiment. The researcher asked them questions from a post-experiment questionnaire.

### 2.5 Data analysis

The data were analyzed using RStudio 2023.09.0 + 463 (RStudio Team, 2020). To test our hypotheses, we performed mixed-effect regression models using lme4 package (Bates et al., 2015). In each model, participants were modeled as random intercepts. The values of *p* for each predictor were obtained via lmerTest package (Kuznetsova et al., 2017). An *a priori* significance level was decided to be *p* < 0.05.

#### 2.5.1 Data pre-processing

##### 2.5.1.1 Data cleaning

After data collection, we removed 12 rebus puzzles from the subsequent analysis owing to unsuccessful encryption, possible alternative solutions, or high variability in the forms of the target expression. Throughout the experiment, we presented participants with the correct solutions asking them to check theirs and to report whether they were the same (self-checking procedure). After data collection, the three experimenters independently verified the participants’ solutions, the results of which were included in the subsequent data analyses. The evaluations were highly consistent between the experts: Expert 1 vs. Expert 2 = 96%, Expert 1 vs. Expert 3 = 96%, Expert 2 vs. Expert 3 = 93% (mean = 96%). The experts discussed questionable trials until consensus was reached. Trials in which participants assessed their incorrect solutions as correct (269 trials, 3% out of all) or their correct solutions as incorrect (58 trials, <1% out of all) were excluded from the analysis. We also removed 16 trials (<1% out of all) in which participants indicated that they had the correct solution when they had none. If participants did not solve the problem correctly, we asked them whether they were familiar with the common expression that served as the solution. Trials in which participants reported not knowing the target expressions were excluded from the analysis (343 trials, 4% out of all). Three participants waited until the deadline to submit solutions for each trial. Their data were excluded from the analysis of solution time.

##### 2.5.1.2 Derived variables

Our main hypothesis concerns a shift in problems’ subjective difficulty. To assess the magnitude of this shift, we computed a new variable by subtracting the retrospective difficulty rating (dif2, measured after solution generation, or dif3, measured after solution presentation) from the initial rating (dif1). Because we measured the difficulty ratings at three stages, we calculated two new variables: (1) the difficulty shift for generated solutions (both correct and incorrect) was calculated as dif1 − dif2 and (2) the difficulty shift for the presented solutions (both incorrect and no solutions) was calculated as dif1 − dif3.

## 3 Results

### 3.1 Descriptive statistics

Participants had correct solutions in 4,460 (58%) trials and incorrect solutions in 1,741 (23%) trials. The average solution times for correct and incorrect solutions were 7.10 s (SD = 6.62) and 17.3 s (SD = 10.5), respectively. Table 1 shows the average subjective ratings across solution types. We used two sets of stimuli in our study assuming that they are equivalent in terms of difficulty. To test this assumption, we compared the both sets’ average accuracy and solution times aggregated by participants and found no significant differences neither in accuracy [M1 = 0.59(0.49), M2 = 0.58(0.49), *t*(155) = 0.732, *p* = 0.466] nor in solution times for correct solutions [M1 = 12.20(7.33), M2 = 12.40(7.50), *t*(146) = −0.638, *p* = 0.525].

**Table 1:** Mean subjective ratings across solution types.

Solution type	Initial difficulty M(SD)	Retrospective difficulty (generated solutions) M(SD)	Retrospective difficulty (presented solutions) M(SD)	Post-solution-generation Aha M(SD)	Post-solution-presentation Aha M(SD)	Confidence M(SD)	Likability M(SD)
Correct	3.45(1.97)	2.80(1.60)	-	3.67(1.83)	-	6.00(1.47)	4.88(1.58)
Incorrect	4.63(1.54)	4.53(1.65)	4.70(1.56)	2.66(1.62)	4.59(1.88)	2.98(1.97)	4.79(1.75)
No solution	5.54(1.13)	-	5.55(1.43)	-	4.47(2.01)	-	4.68(1.95)

### 3.2 Initial subjective difficulty rating as predictors of accuracy

**Figure 2:**
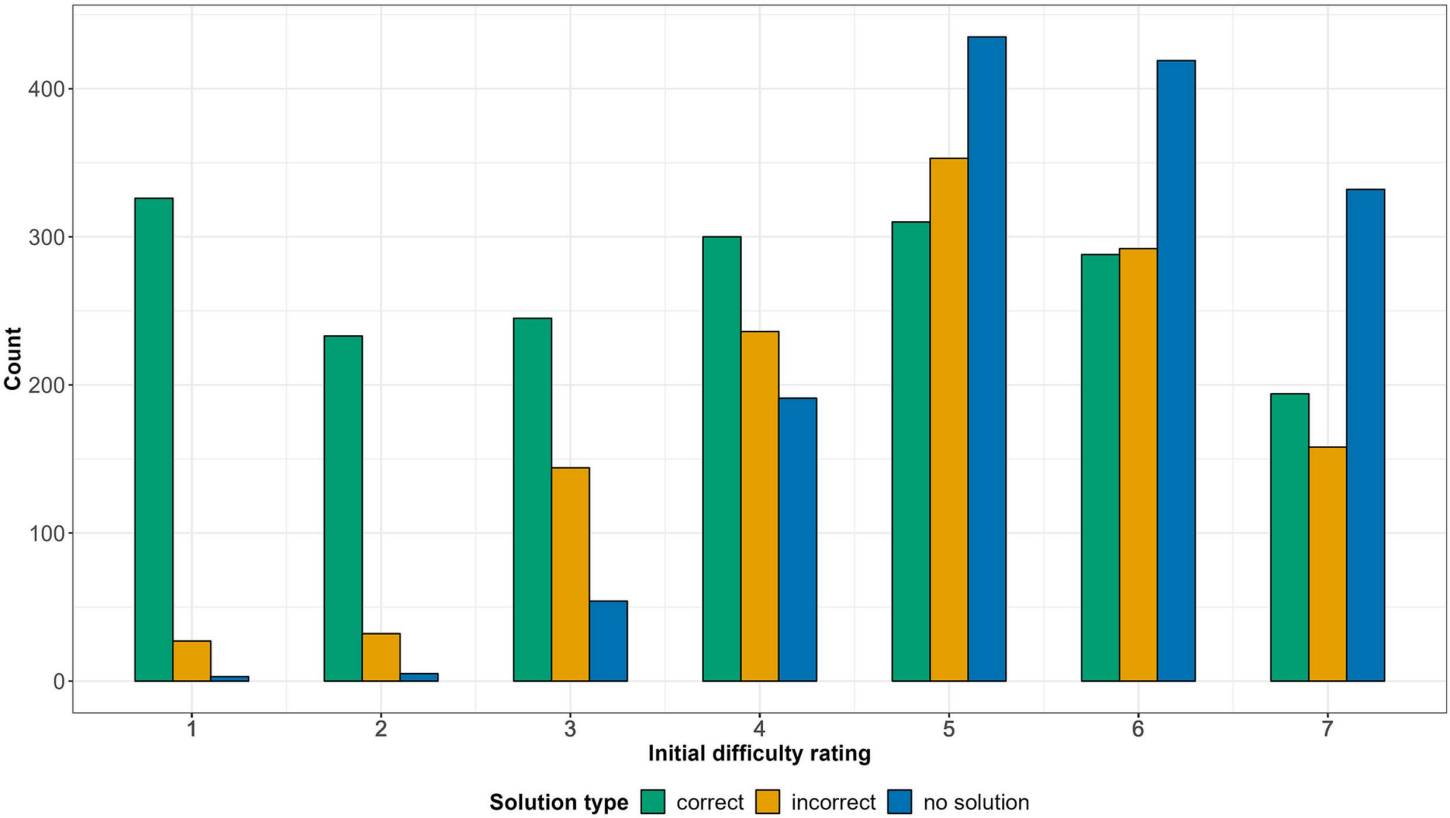
Frequencies of different initial difficulty ratings across different solution types.

To test whether the initial difficulty rating predicted accuracy, we used a mixed-effect linear regression model with the initial difficulty rating as an outcome variable and solution type (correct/incorrect/no solution) as a fixed effect. Participants were modeled as random intercepts. We removed all solutions generated before providing the rating (the first 5 s of a trial) and solutions given within 1 s after providing the initial difficulty rating from the analysis. Figure 2 displays the frequencies of different initial difficulty ratings across solution types. The model was significant and revealed that the higher the initial difficulty ratings, the higher the probability of both incorrect and no solutions (Table 2).

**Table 2:** The results of the regression model with initial difficulty rating as a dependent variable and solution type as a fixed effect.

Predictor	Beta	SE	*t* value	*p* value
Intercept	4.00	0.07	59.89	**<0.001**
Incorrect solution	0.95	0.05	17.26	**<0.001**
No solution	1.53	0.05	29.07	**<0.001**

Significant predictors (*p* < 0.05) are shown in bold.

### 3.3 Prospective subjective difficulty and the Aha! experience as predictors of solution time of correct solutions

**Figure 3:**
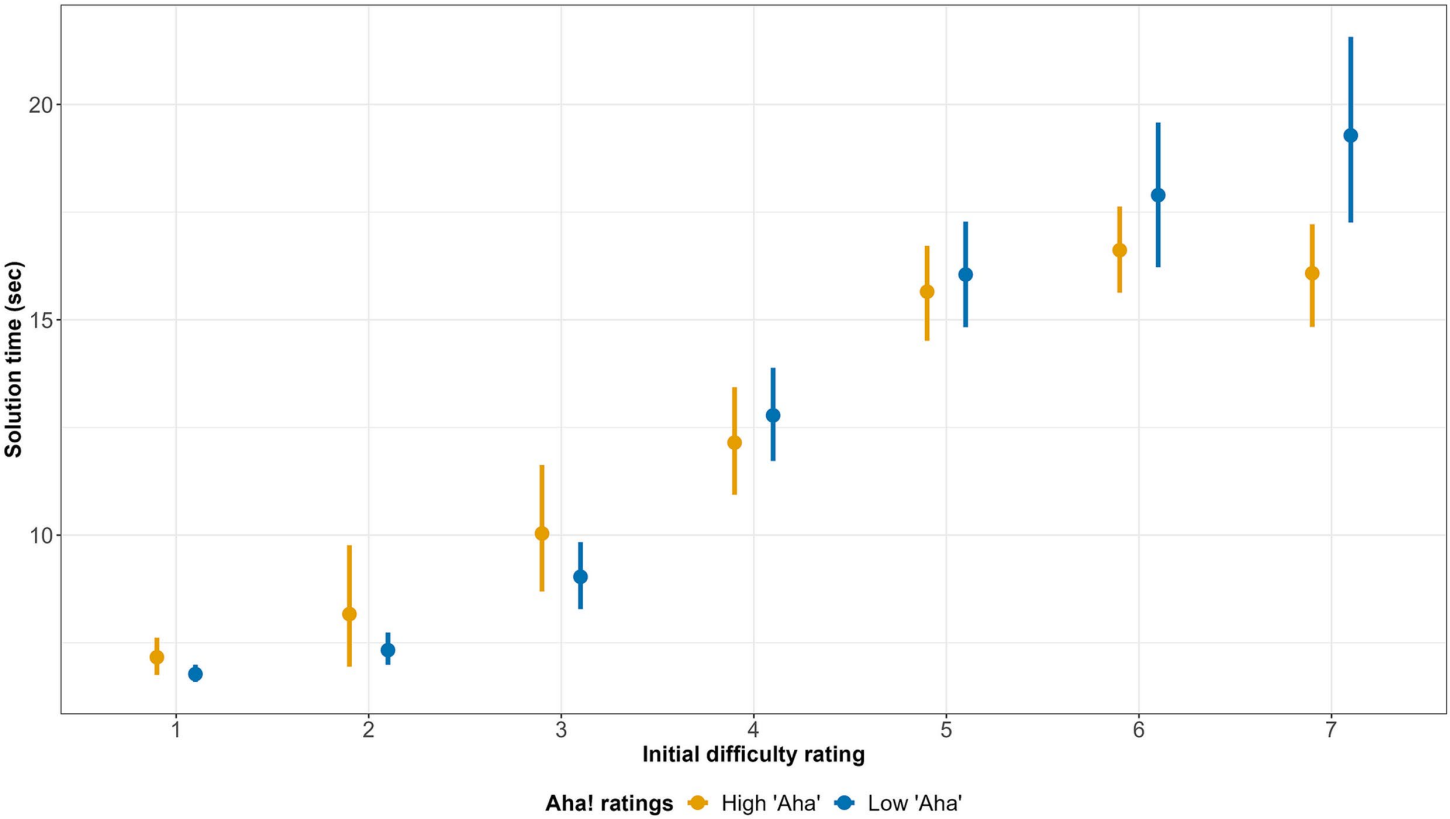
Solution times by initial difficulty ratings (only correct solutions). The binary “High Aha”—“Low Aha” variable was derived from the original seven-point Aha! experience rating (The bars refer to 95% CI).

We assumed that the initial difficulty rating would predict the solution time of correctly solved problems: the higher the initial ratings the more time the solver would spend to find the solution. But the strength of this correlation would decrease with an increase in Aha! experience ratings. In other words, we expected a negative effect of the interaction between initial difficulty ratings and the Aha! experience ratings on solution time. Only correct solutions were included in this analysis. Again, the solutions generated within the first 6 s of the trial were removed from the analysis as they were submitted before or immediately after the initial difficulty rating. We ran a mixed-effect linear regression with solution time as a dependent variable and the initial difficulty rating, the Aha! experience rating, and their interaction as fixed effects. The complete model revealed a significant positive effect of the initial difficulty rating and the negative effect of its interaction with the Aha! experience ratings (Table 3). This result indicates that the harder the problem seemed to participants, the more time they needed to find the correct solution. However, for the problems evaluated as difficult, faster solutions were associated with higher Aha! experience scores (see Figure 3). Figure 3 shows the average solution times for different initial difficulty ratings. For the ease of visualization Aha! experience ratings were transformed into a binary variable with “High Aha!” solutions scored as those higher than 4 and “Low Aha!” solutions as those lower than 4 on the seven-point scale (the “4” rating did not fall into either categories).

**Table 3:** The results of the regression model with solution time as a dependent variable and initial difficulty rating, Aha! experience rating, and their interaction as fixed effects.

Predictor	Beta	SE	*t* value	*p* value
Intercept	2.63	0.74	3.571	**< 0.001**
Initial difficulty	2.56	0.18	14.248	**< 0.001**
Aha	0.33	0.20	1.680	0.093
Initial difficulty*Aha	−0.10	0.04	−2.485	**0.013**

Significant predictors (*p* < 0.05) are shown in bold.

### 3.4 Subjective difficulty shift and the Aha! experience

Our main hypothesis presumed that the difference between the retrospective and initial difficulty ratings would be higher for the trials with the higher Aha! experience. This would indicate a sudden and significant change in processing fluency associated with the Aha! experience. We performed three similar models for each type of solution. The post-solution-generation Aha! experience was used as a main predictor in the first model for correct solutions, while the post-solution-presentation Aha! experience was used in the two latter models for incorrect solutions and no-solution trials. The models for correct and incorrect solutions also included solution time and its interaction with Aha!-ratings as fixed effects. The dependent variable in the three models was the difference between the initial difficulty ratings and the retrospective one (the shift in difficulty).

**Figure 4:**
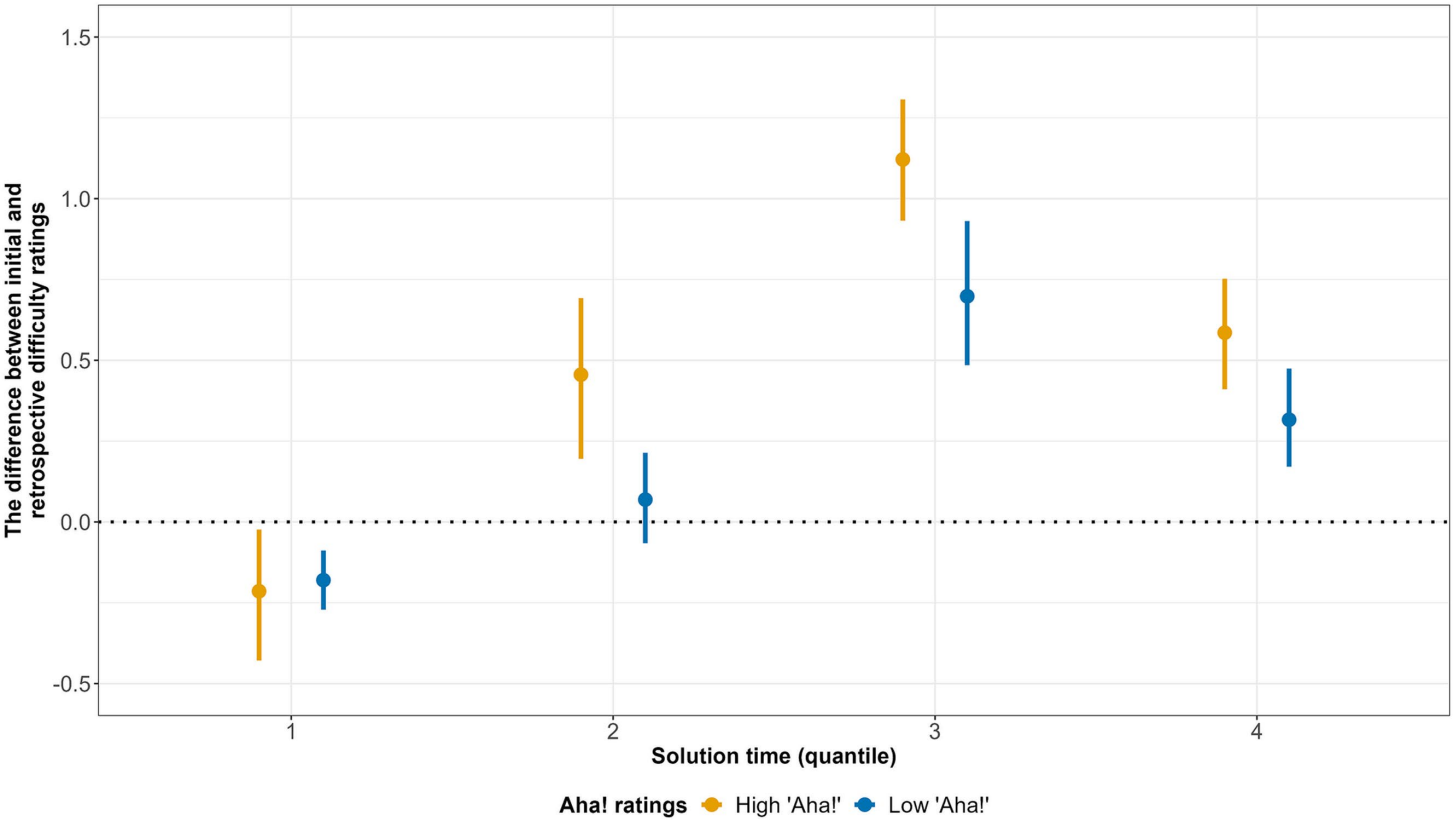
The average difference between initial and retrospective difficulty ratings by the Aha! experience rating and solution time (only correct solutions). Solution Time variable was derived by splitting the original Solution Time variable into quartiles (Bars refer to 95% CI).

The first model for correct solutions had the Aha! experience ratings, solution time, and their interaction as fixed effects. We removed solutions generated within the first 6 s of a trial from the analysis. The model showed significant positive effects of the Aha! ratings and solution times, indicating that the subjective difficulty of the problem drops more dramatically when the solution is accompanied with a stronger Aha! experience (see Table 4). The bigger shift in subjective difficulty is also associated with longer solutions, but the effect is rather small. The interaction between the two measures was not significant. Figure 4 illustrates the results of the model by showing the average shift in the difficulty ratings depending on the Aha! Ratings. For visualization reasons, the binary Aha! variable was derived by assigning “Low Aha” and “High Aha” labels to the values less and more than 4, respectively. The solution time variable was split into quartiles.

**Table 4:** The results of the regression model with the shift in subjective difficulty as a dependent variable and Aha! experience rating, solution times, and their interaction as fixed effects (only correct solutions trials).

Predictor	Beta	SE	*t* value	*p* value
Intercept	−0.50	0.16	−3.226	**< 0.001**
post-solution-generation Aha	0.18	0.04	4.911	**< 0.001**
Solution time	0.03	0.01	2.707	**0.007**
Aha*Solution time	−0.003	0.002	−1.380	0.168

Significant predictors (*p* < 0.05) are shown in bold.

The second model was performed only for generated incorrect solutions. The fixed effects were post-solution-presentation Aha! experience and solution time. The outcome variable was a shift in difficulty, calculated as the retrospective difficulty rating subtracted from the initial difficulty rating given after the presentation of the correct solution. The model revealed no significant effects (Table 5).

**Table 5:** The results of the regression model with the shift in subjective difficulty as a dependent variable and Aha! experience rating, solution time, and their interaction as fixed effects (only incorrect solutions trials).

Predictor	Beta	SE	*t* value	*p* value
Intercept	−0.28	0.25	−1.063	0.288
Post-solution-presentation Aha	0.03	0.08	0.340	0.734
Solution time	0.009	0.01	0.863	0.388
Aha*Solution time	0.001	0.004	0.340	0.734

Significant predictors (*p* < 0.05) are shown in bold.

**Figure 5:**
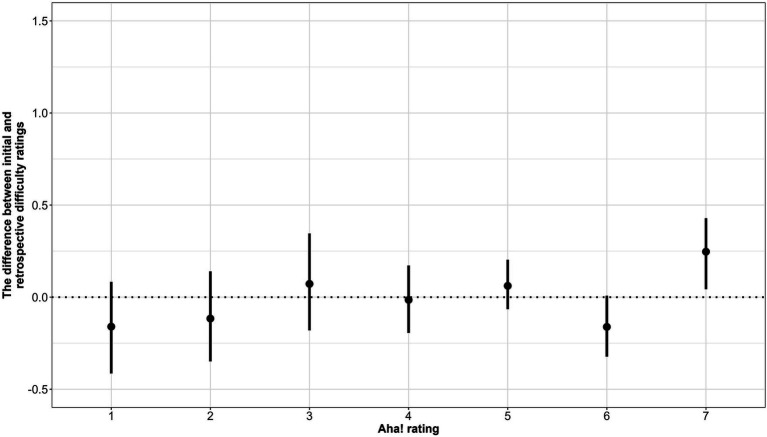
The average difference between initial and retrospective difficulty ratings by the Aha! experience rating (only trials without generated solutions; Bars refer to 95% CI).

The trials with no solutions were included in the third analysis. The third model was similar to the previous one but did not include solution time as a predictor. This model revealed a significant positive effect of the post-solution-presentation Aha! experience (Table 6); that is, the stronger the Aha! experience, the bigger the drop in the subjective difficulty of the problem. The average shift of the difficulty ratings for each post-solution-presentation Aha! experience rating is depicted in Figure 5. As we can see, the trials with the highest Aha! ratings contributed significantly to the aforementioned effect.

**Table 6:** The results of the regression model with the shift in subjective difficulty as a dependent variable and Aha! experience rating as a fixed effect (only no solutions trials).

Predictor	Beta	SE	*t* value	*p* value
Intercept	−0.25	0.11	−2.252	**0.025**
Post-solution-presentation Aha	0.05	0.02	2.527	**0.012**

Significant predictors (*p* < 0.05) are shown in bold.

### 3.5 Relationship between the Aha! experience, confidence, problem likability, and accuracy

Apart from the main hypotheses, we put forward several auxiliary hypotheses, which we derived from the previous research on the Aha! experience. Reportedly, the Aha! experience is often associated with confidence, problem likeability, and accuracy. We performed two mixed-effect linear regression models to investigate these relations. The first one was run on the trials that had the post-solution-generation Aha! experience ratings (i.e., correct or incorrect solutions generated by participants). The Aha! rating was the dependent variable and the fixed effects were the solution type (correct/incorrect), confidence rating, and likability rating. The model revealed a positive correlation between the Aha! experience and confidence and between the Aha! experience and likability (Table 7). Correct solutions were accompanied with a stronger Aha! experience than incorrect ones. As confidence is usually strongly associated with the actual accuracy of the solutions, we tested our model for multicollinearity and found a moderate correlation [variance inflation factor (VIF) = 1.89].

**Table 7:** The results of the regression model with the Aha! experience rating as a dependent variable and confidence rating, likability rating, and solution type (correct vs. incorrect) as fixed effects.

Predictor	Beta	SE	*t* value	*p* value
Intercept	0.45	0.11	4.068	**<0.001**
Confidence	0.17	0.01	14.780	**<0.001**
Correct solution	0.49	0.06	8.690	**<0.001**
Likability	0.35	0.01	27.667	**<0.001**

Significant predictors (*p* < 0.05) are shown in bold.

The second model included trials with the post-solution-presentation Aha! experience ratings (i.e., incorrect solutions and no solutions). The Aha! rating was the dependent variable, while the likability rating and solution type (incorrect/no solution) were the fixed effects. The model revealed a positive effect of likability but no effect of solution type (see Table 8).

**Table 8:** The results of the regression model with the Aha! experience rating as a dependent variable, likability ratings and solution type (no solutions vs. incorrect) as fixed effects.

Predictor	Beta	SE	*t* value	*p* value
Intercept	2.02	0.11	17.982	**<0.001**
Incorrect solution	0.02	0.06	0.421	0.674
Likability	0.53	0.02	34.479	**<0.001**

Significant predictors (*p* < 0.05) are shown in bold.

### 3.6 Additional analysis

An Aha! experience is often considered a heuristic that people rely on to select the best idea. However, sometimes there are false insights—incorrect solutions accompanied by the Aha! experience. Our data provide an opportunity to examine this phenomenon from an interesting perspective, namely, the degree to which people are ready to accept that their solution was wrong when it was or was not accompanied by an Aha! experience. In our experiment, participants had to compare their solutions to the correct ones and press certain buttons when they were correct or incorrect. In some trials, participants assessed their solutions as correct even if they differed from the presented correct solutions. We decided to analyze whether such self-check errors are associated with a stronger Aha! experience. We took the trials where participants assessed their incorrect solutions as correct and performed a mixed-effect regression model with the Aha! experience ratings as an outcome variable and the self-check error as a fixed effect. The model was significant and revealed the positive effect of the Aha! experience ratings, which indicates that Aha! experience ratings were higher for the trials where participants refused to accept that their solutions were wrong (M_no self-check error_ = 2.44, M_self-check error_ = 3.29, *β* = 0.80, SE = 0.11, *t* = 7.018, *p* < 0.001).

## 4 Discussion

The purpose of this study was to investigate the relationship between the intensity of the Aha! experience and the subjective difficulty ratings of the problem before and after its solution. We hypothesized that the Aha! experience is the result of the shift in the processing fluency that appears due to representational change from an incoherent representation of the problem to a coherent one, resulting in the solution’s retrieval with unexpected ease. Since metacognition theories suggest that processing fluency underlies the assessment of a problem’s subjective difficulty (Ackerman, 2019), we expected that higher Aha! experience ratings would correspond to a greater difference in the subjective difficulty ratings between the initial assessment (5 s after the problem presentation) and the retrospective assessment (after generating a correct solution or after presenting the correct solution).

Based on previous research on the predictability of initial solvability ratings (Topolinski and Strack, 2008; Burton et al., 2023), we expected that initial subjective difficulty ratings given within the first 5 s would predict the probability of a correct solution and the time required to do so. We also expected that higher Aha! experience ratings would indicate the suddenness of the solution, which would manifest in the decrease of the correlation between the initial subjective difficulty ratings and solution time.

To examine our assumptions, we developed a new set of rebus puzzles in Russian that are solved by restructuring or relaxing constraints. Usually, words’ visual–spatial characteristics (e.g., color and font size, spatial arrangement) are irrelevant to their meaning. However, in rebuses, people need to use these characteristics to figure out the meaning of common expressions. Previous studies showed that the rebus solution process is often accompanied by an Aha! experience (Salvi et al., 2016b; Threadgold et al., 2018). An analysis of our results showed that the developed rebuses vary in difficulty level (the probability of a correct solution varies from 100 to 5%). The average solution time was 7.10 s (SD = 6.62), with the maximum solution time being 30 s. These results are similar to those of Salvi et al. (2016b) and Threadgold et al. (2018), where the average time to solve puzzles was 4.07 and 12.55 s, respectively.

### 4.1 Relationship between subjective difficulty ratings, the intensity of the Aha! experience, and the objective difficulty of the problem (accuracy and solution time)

We analyzed the subjective difficulty ratings, which were measured three times: 5 s after the rebus presentation (the first, initial rating), immediately after the solution generation (the second rating), and, in case the solution was omitted or incorrect, after the correct solution presentation (the third rating). First, we analyzed whether initial difficulty ratings predict the probability of the correct solution, excluding observations with a solution time faster than 6 s since participants could have already found the solution before they rated the problem’s difficulty. The analysis showed that the initial subjective difficulty ratings correlated with the problem’s objective difficulty: the higher the subjective difficulty rating, the lower the probability of the correct solution within the next 24 s. Thus, we can conclude that participants have at least partial metacognitive access to the unfolding solution process, which allows them to adequately assess the probability of solving a problem (i.e., its difficulty) before the solution emerges into consciousness. The analysis of the relationship between the initial subjective difficulty rating and correct solution time also confirmed the above conclusion. The higher the initial difficulty ratings, the greater average time taken to find the solution. According to our hypothesis, metacognitive access had to be observed in non-insightful solutions; that is, those with the low Aha! experience ratings. We hypothesized that insightful solutions (those with high Aha! experience ratings), would be more unexpected for participants, so the correlation between the initial difficulty ratings and solution time would decrease. Our results confirmed these assumptions as we found the negative interaction effect of the initial difficulty and Aha! experience ratings on the solution time. In other words, for problems with the same subjective difficulty, insightful solutions result in faster answers than do non-insightful ones.

Our results are consistent with model of Dubey et al. (2021), in which the intensity of the Aha! experience reflects a positive prediction error regarding solution time. This result is also consistent with our assumption that the intensity of the Aha! experience indicates the magnitude of the shift between the actual and expected processing fluency. According to our second hypothesis, this shift would result in a more significant decrease in the retrospective subjective difficulty ratings. Therefore, we expected that higher Aha! experience ratings would correspond to a greater difference between the initial and retrospective subjective difficulty ratings. We built three regression models to examine this hypothesis: (1) for the post-solution-generation Aha! experience in trials with generated correct solutions, (2) for the post-solution-presentation Aha! experience in trials when participants were presented with correct solutions after generating incorrect ones, and (3) for the post-solution-presentation Aha! experience in trials where participants omitted solutions and were presented with the correct one. Our hypothesis was confirmed by the results of the first and the third regression models. If participants independently found the correct solution to the rebus, their Aha! experience ratings positively correlated with a decrease in the retrospective subjective difficulty rating. Similarly, if participants did not find any solution and saw the correct one, their post-solution-presentation Aha! experience rating positively correlated with a decrease in the subjective difficulty rating (it is worth noting that the highest ratings of the Aha! contribute to the effect the most).

The latter result is of special interest as we might have expected that in case participants could not find the solution, they would increase the problem’s retrospective difficulty rating. However, the strong Aha! experience at the moment of understanding the presented solution and the high processing fluency of a new problem representation presumably corresponding to it leads them to think that the problem was not so difficult. This could explain cases when participants, after learning the correct solution to an insight problem, exclaim “Why did not I think of that before?” (See Gick and Lockhart, 1995, p.199 for details).

On the other hand, we did not find a correlation between the shift in subjective difficulty and the Aha! ratings when solutions were presented after incorrect solutions. Perhaps the false idea of the solution appeared in the rebuses and provoked the activation of an irrelevant but frequent common expression, making them seem solvable. Consequently, the initial difficulty rating was not so different from the one participants gave after finding out the correct solution.

Our results are consistent with and complement those of previous studies with CRAT (Stuyck et al., 2021; Moroshkina et al., 2022). Moroshkina et al. (2022) studied the retrospective subjective difficulty of problems using a binary scale (difficult/easy) and found that the likelihood of judging the unsolved problem to be difficult decreased if the participant had the post-solution-presentation Aha! experience. This result was obtained while controlling for objective difficulty, calculated as the probability of solving the problem and aggregated over the entire sample. In our study with subjective difficulty ratings measured before and after the finding a solution we managed to show that the magnitude of the shift between the initial and final difficulty ratings was correlated with the intensity of the post-solution-generation Aha! experience. We obtained a similar result for the post-solution-presentation Aha! experience when the solution was omitted. It should be noted that if participants reported that they did not know the encrypted expression, the trial was excluded from the analysis. Thus, the discovered correlation could not appear due to the contribution of incomprehensible solutions.

The study by Stuyck et al. (2021) is the only one we know of to have investigated the initial and final subjective difficulty ratings in relation to the solution strategy (insight vs. non-insight). They hypothesized that the final difficulty rating would indicate the fluency of solution retrieval and, therefore, would be lower in the case of insight solutions compared to non-insight ones. However, they did not find the expected correlation. Our hypothesis differs from that of Stuyck et al. (2021) as we propose that the Aha! experience does not reflect the absolute level of processing fluency but the relative level (the actual compared to the expected; see also Whittlesea and Williams, 2001). Therefore, we expected the Aha! experience ratings to positively correlate not with the final difficulty rating itself but rather with the magnitude of the shift in the final difficulty rating relative to the initial one. Our results confirmed this assumption, as did the results of the study by Stuyck et al. (2021), which we independently analyzed (Moroshkina et al., 2022) based on their OSF database (see text footnote 1). Thus, we obtained important results providing evidence that the positive prediction error in processing fluency (when the actual processing fluency exceeds the expected one) is the source of the Aha! experience and its intensity. This error can occur due to restructuring when the initial incoherent representation changes to a new, more coherent one and the solution is retrieved unexpectedly easily and quickly. However, further research is necessary to examine this assumption on different problem sets with varying types and objective difficulty.

### 4.2 Phenomenology of insight in solving rebus puzzles

As we develop a new problem set for the study of insight in Russian speakers, we put forward several auxiliary hypotheses to examine the similarity of the phenomenology of insightful solutions in our research with the results of previous works. We tested three effects that explored (1) the association of the Aha! experience ratings with confidence in the solution (the Aha!-confidence effect), (2) the association of the Aha! experience rating with the correctness of the solution (the Aha!-accuracy effect), and (3) the association of the Aha! experience after the solution generation and solution presentation with the likability of the rebus idea (the Aha!-likability effect). All auxiliary hypotheses were confirmed. The results of the analysis showed that confidence in the solution was positively correlated with the Aha! experience ratings both for correct and incorrect solutions. This result is consistent with the previous data on magic trick materials (Danek and Wiley, 2017) and CRAT (Stuyck et al., 2021; Moroshkina et al., 2022) and favors the Eureka heuristic (Laukkonen et al., 2023). Obtaining the Aha!-confidence effect not only for correct solutions but also for incorrect ones corresponds to the processing fluency account (Topolinski and Reber, 2010). According to this account, both Aha! and confidence are triggered by an increase in processing fluency, which serves as a cue for the inference of metacognitive judgments and thus can sometimes lead to errors (i.e., false insights). To the best of our knowledge, we are the first study to obtain this result on rebus puzzles, since in previous works on similar material, the effect was either not obtained (Threadgold et al., 2018) or was not examined (MacGregor and Cunningham, 2008; Salvi et al., 2016a,b).

We also found the Aha!-accuracy effect: Aha! experience ratings were, on average, higher for correct solutions than for incorrect ones. This result is in line with the findings of previous studies (Danek et al., 2014; Zedelius and Schooler, 2015; Webb et al., 2016; Laukkonen et al., 2021; Moroshkina et al., 2022), including those with rebus puzzles (Salvi et al., 2016a,b; Threadgold et al., 2018), although the Aha!-accuracy effect was not always found (Stuyck et al., 2021; Strickland et al., 2022).

In our experimental procedure, participants self-checked their solutions, comparing them with the presented correct one. After data collection, three experimenters independently verified the data and found a relatively small percentage of trials in which participants made self-check errors (They judged 269 incorrect answers as correct and 58 correct answers as incorrect ones). We excluded these observations from the main analysis but performed an additional analysis as we were interested in whether the probability of a self-check error was related to the Aha! experience at the moment of solution generation. The results showed that the Aha! ratings were higher for the trials where participants refused to accept that their incorrect solutions were wrong; thus, we can presume that the Aha! experience can be used by solvers as the accuracy heuristic not only in the absence of objective feedback but also despite it. In other words, participants who experiencеd the strong Aha! could not notice the difference between their own solutions and the presented solutions or they could believe that their own solutions were better (more correct) than the presented ones.

In part, this result can be aligned with the findings of Hedne et al. (2016) on magic tricks. Participants were asked to suggest solutions for how the trick was done and to then choose from four possible solutions (with the only one correct) provided by the experimenters. It turned out that if the participants experienced a false insight (i.e., an incorrect idea accompanied by an Aha! experience), they more often selected the one alternative that was most similar to their own solution and not the correct one. If their incorrect idea was not accompanied by an Aha! experience, they either repeated or changed their solution to another one with a 50/50 probability.

Finally, in line with our previous work (Moroshkina et al., 2022), we found that the Aha! experience ratings after both solution generation or presentation were correlated with the likability of the rebus idea. Previous studies have highlighted that positive emotions are one of the components of the Aha! experience (Danek et al., 2014; Shen et al., 2016; Danek and Wiley, 2017). The processing fluency hypothesis (Topolinski and Reber, 2010), as well as the positive metacognitive prediction error model (Dubey et al., 2021), assign an important role to positive affect as part of the Aha! experience. Presumably, it serves as an internal reward and helps consolidate an insightful solution in memory (Kizilirmak et al., 2019; Kizilirmak and Becker, 2023). Our results suggest that the positive emotions associated with an Aha! experience may be attributed to the rebus likability ratings, explaining why solving such puzzles as rebuses, crosswords, charades, etc. often becomes a hobby or a form of entertainment for people in their free time.

## 5 Limitations

The Aha! experience is considered a multi-dimensional phenomenon (Danek et al., 2014; Danek and Wiley, 2017). In our study, we examined the association of the Aha! experience and metacognitive prediction error with the problem’s subjective difficulty while measuring only the overall Aha! experience ratings on a seven-point scale. However, further research is needed to test the relationship of each component of the Aha! experience (e.g., suddenness, surprise, and confidence) with the metacognitive prediction error and reduction of subjective difficulty.

Moreover, it is worth noting that metacognitive monitoring itself could be studied using various measures (e.g., judgment of solvability, task difficulty, mental efforts and warmth ratings). Our study established an association between the Aha! experience and metacognitive prediction error with regard to solution time and the problem’s initial subjective difficulty rating. Previous studies have demonstrated the correlation between the Aha! experience and warmth rating patterns (Kizilirmak et al., 2018; Laukkonen et al., 2021). Future research should be directed at understanding the extent to which different metacognitive assessments are related to each other and whether they reflect the single process of metacognitive monitoring or each of them has its own specificity.

We also measured metacognitive assessment (the subjective difficulty of the problem) only at the beginning of the solution process and after obtaining the solution. In various trials, depending on the solution time, an interval between two ratings could vary from 1 to 25 s. Thus, it seems reasonable that the solution prediction is adjusted during the problem-solving progress. It remains unclear then, how often it is updated and how it relates to the subsequent Aha! experience.

## 6 Conclusion

Our study aimed to examine the relationship between the intensity of the Aha! experience and the subjective difficulty ratings of the problem before and after its solution. Drawing from the approaches of Topolinski and Reber (2010) and Dubey et al. (2021), we hypothesized that the probability and intensity of the Aha! experience would be closely related to the metacognitive assessments of the problem itself (initial and retrospective difficulty ratings). Our first hypothesis addressed the idea that the Aha! experience is associated with a metacognitive prediction error. The Aha! experience arises when the time taken to solve a problem turns out to be less than what was predicted based on the processing fluency of the initial problem representation. We hypothesized that higher Aha! experience ratings would indicate more sudden solutions, manifesting in a reduced correlation between the initial difficulty ratings and solution times. The general result indicated that the harder the problem seemed to the participants, the more time they needed to find the correct solution. However, for the problems evaluated as difficult, faster solutions were associated with higher Aha! experience ratings, supporting the metacognitive prediction error approach.

The second hypothesis is a logical extension of the first: we expected that the difference between the initial difficulty ratings and the retrospective ones would be higher for the trials with the higher Aha! experience. We proposed this hypothesis based on the premise that if the Aha! experience is associated with a disruption of expectations regarding the problem’s difficulty (how quickly and easily it can be solved), one would anticipate a greater reduction in subjective difficulty ratings for insightful solutions compared to non-insightful ones. Our hypothesis was confirmed: in cases when participants found the solutions themselves, the Aha! experience was positively correlated with a shift between the initial and retrospective subjective difficulty ratings. Similarly, if participants did not find any solution and saw the correct one, their post-solution-presentation Aha! experience rating was positively correlated with a decrease in the subjective difficulty rating.

We also proposed three auxiliary hypotheses based on numerous studies that have shown that the Aha! experience correlates with solution accuracy (Salvi et al., 2016a,b; Threadgold et al., 2018), positive emotions (Danek et al., 2014; Shen et al., 2016; Danek and Wiley, 2017; Savinova and Korovkin, 2022), and positive evaluation of the problem (likability; Moroshkina et al., 2022). Our results are consistent with those of previous studies. We found the Aha!-confidence, Aha!-accuracy, and Aha!-likability effects. Moreover, we conducted an exploratory analysis that showed that participants who experienced a strong Aha! moment may believe that their solutions are better (more correct) than the presented ones, which is in line with the work of Hedne et al. (2016). In summary, we present results that support both the metacognitive prediction error and processing fluency approaches.

## Data availability statement

The datasets presented in this study can be found in online repositories. The names of the repository/repositories and accession number(s) can be found below: https://osf.io/a7b3z/.

## Ethics statement

The studies involving humans were approved by Ethical Committee of Saint Petersburg Psychological Society, #IRB00012426. The studies were conducted in accordance with the local legislation and institutional requirements. The participants provided their written informed consent to participate in this study.

## Author contributions

NM: Conceptualization, Methodology, Supervision, Writing – original draft. EP: Data curation, Formal Analysis, Investigation, Writing – original draft. AA: Conceptualization, Formal Analysis, Methodology, Software, Visualization, Writing – original draft. VG: Conceptualization, Methodology, Writing – review & editing. OL: Investigation, Methodology, Writing – review & editing.

## Footnotes

^1^https://osf.io/5jtxg/

^2^https://osf.io/a7b3z/

^3^pavlovia.org
