# Supplementary material for: The Aha! experience is associated with a drop in the perceived difficulty of the problem
Source: Front Psychol. 2024 Jan 23;15:1314531. doi: 10.3389/fpsyg.2024.1314531 (PMC10844378; doi:10.3389/fpsyg.2024.1314531)
Supplement: Supplementary file 1 [file Data_Sheet_1.docx]

Supplementary Material 1

*Instructions*

Thank you for agreeing to participate in the study! Please read carefully the instructions below, and if you have any questions, ask the experimenter.

Your task is to solve as many rebuses as possible. Each rebus contains a common expression in the Russian language. For example, in this rebus (note: rebus is presented on the screen), the expression “сидеть на двух стульях” (eng: to sit on two chairs) is encrypted. Common expressions consist of two or more words. When entering your answer, please try to write the expression exactly as you remember it.

There will be a total of 58 rebuses. Initially, each rebus will appear for 5 seconds, and you need to assess its difficulty on a 7-point scale. After that, the rebus will be presented again, and you will have 25 seconds to solve it. As soon as you understand that you have found a solution to the rebus, press the spacebar and type your answer in the window that appears on the screen. You can do this at any time while you see the rebus on the screen. When 25 seconds end, the rebus will disappear, and a window for entering the answer will appear automatically.

If you couldn't solve the rebus, please leave the window blank. After that, we will show you the correct answer and ask you to check whether it matches the one you provided. When comparing your solution to the presented one, answer “yes” (the answer matches the correct one) only if, when entering your answer, you meant the same expression that is shown on the screen (minor deviations are possible).

After that, we will ask you to answer some questions and assess the difficulty of this rebus again. If everything is clear, let's practice. You will have three practice rebuses. To do this, press the right arrow.

*An English translation is provided. Instructions were presented in Russian. See the original instructions at OSF:* <https://osf.io/a7b3z/>
